# The uncanny return of the race concept

**DOI:** 10.3389/fnhum.2014.00836

**Published:** 2014-11-04

**Authors:** Andreas Heinz, Daniel J. Müller, Sören Krach, Maurice Cabanis, Ulrike P. Kluge

**Affiliations:** ^1^Department of Psychiatry and Psychotherapy, Charité—University Medicine BerlinBerlin, Germany; ^2^Centre for Addiction and Mental Health, Department of Psychiatry, University of TorontoToronto, ON, Canada; ^3^Department of Child and Adolescent Psychiatry, Philipps-University MarburgMarburg, Germany; ^4^Center for Mental Health, Klinikum StuttgartStuttgart, Germany

**Keywords:** cultural neuroscience, racial classification, intelligence (IQ), racism, psychiatry

## Abstract

The aim of this *Hypothesis and Theory* is to question the recently increasing use of the “race” concept in contemporary genetic, psychiatric, neuroscience as well as social studies. We discuss “race” and related terms used to assign individuals to distinct groups and caution that also concepts such as “ethnicity” or “culture” unduly neglect diversity. We suggest that one factor contributing to the dangerous nature of the “race” concept is that it is based on a mixture of traditional stereotypes about “physiognomy”, which are deeply imbued by colonial traditions. Furthermore, the social impact of “race classifications” will be critically reflected. We then examine current ways to apply the term “culture” and caution that while originally derived from a fundamentally different background, “culture” is all too often used as a proxy for “race”, particularly when referring to the population of a certain national state or wider region. When used in such contexts, suggesting that all inhabitants of a geographical or political unit belong to a certain “culture” tends to ignore diversity and to suggest a homogeneity, which consciously or unconsciously appears to extend into the realm of biological similarities and differences. Finally, we discuss alternative approaches and their respective relevance to biological and cultural studies.

## Introduction

In 1996, the Association of American Physical Anthropologists (AAPA) issued a statement on putatively biological aspects of “race” that rejected the concept as having no scientific utility (Association of American Physical Anthropologists, 1996). Nevertheless, about a decade ago, the Society for Neuroscience (SfN) asked all their conference participants to classifying themselves as “belonging to a certain race such as Caucasian, African American” etc. “Race” classifications were part of mainstream thinking of Apartheid in South Africa; however this SfN procedure was not aimed at reifying such dubious classifications, but rather at promoting participation of minorities in the conference. It had been initiated by advocacy groups who wanted to promote the presence of scientists belonging to “racially” defined social minorities. Obviously, this effort is not motivated by an uptick in 19th century racism, but instead by an attempt to increase diversity: “to keep issues of social inequality in the forefront of such research”, as Blum (2010) concludes. However, this effort has its unintended negative consequences: if social exclusion is to be reduced by promoting participation of subjects of a certain “race”, the concept of “race” itself tends to be reified. Indeed, some organizations for human rights have therefore suggested to replace statements such as “No person shall be favored or disfavored because of sex, parentage, race, language, homeland and origin, faith, or religious or political opinions. No person shall be disfavored because of disability. Nobody can be discriminated against due to his or her race, gender etc.” (Basic Law for the Federal Republic of Germany, Article 3, paragraph 3) by the statement that “racist discrimination is absolutely inacceptable” (Cremer, 2012).

In line with these considerations, Lawrence Blum warned that “protection against unwarranted…racializating of group health differences…[may fail] to capture the historical process of racialization” (Blum, 2010), i.e., it can unintentionally reify the race concept, and thus potentially harm exactly the same subjects who are to be protected by human rights advocacy. The sociologist Robert Miles also “[uses] the concept of racialization to denote a dialectical process by which meaning is attributed to particular biological features of human beings, as a result of which individuals may be assigned to a general category of persons” (Miles, 1989[2003], p. 102). The concept therefore refers to a “process of categorization, a representational process of defining an Other, usually, but not exclusively, somatically” (Miles, 1989[2003], p. 101), thereby “attributing meaning to a real or alleged biological characteristic” (Gupta et al., 2007). Blum states that “unlike classic races, racialized groups exist” (ibid.).

These considerations shed light on a complex and controversial topic, not only in the neurosciences, but likewise in related disciplines such as psychiatry. While there is no doubt that racism exists, the extent to which concepts of “races” promote or harm the scientific process of knowledge production remains an open question. In posing this question, this article wants to distinguish between (1) a biological concept of “race”, which—as discussed in detail below—classifies subjects according to stereotypically perceived phenotypic variations in mutually exclusive “boxes” and assumes that such phenotypic features are genetically fixed and predict a series of further, equally genetically determined characteristics; and (2) the existence of racist discrimination, which operates by such classifications and the associated prejudices. In other words, we argue that although there are no biologically defined “races”, this does not necessarily exclude racism. Indeed, for example in the health care system, people may be treated differently according to attributed “race”, which may allow social exclusion and discrimination to impair access to health care services and treatment (Lee et al., 2001; Wyatt et al., 2003).

Another reason for the increasing use of the “race” concept is genetic research in psychiatry. There is hardly a genetic paper to be published in the realm of biological psychiatry, in which the authors are not requested to identify the “race” of the subjects included in their study. The major concern of reviewers is that the underlying biologic/genetic variation in any examined population is larger when subjects, or their ancestors, come from diverse places in the world. However, does the need for increased homogeneity of study populations scientifically justify the use of traditional, old-fashioned classification terms such as “race” and what are the underlying implications of such classifications? Likewise, in the social sciences there is a longstanding debate on the usage of the often vaguely defined term “culture”, which in many circumstances appears to be used as a proxy for the term “race” or “ethnic” group (Taguieff, 1991; Balibar and Wallerstein, 2011; Kluge and Bostanci, 2012; Martínez Mateo et al., 2012, 2013a,b). Thus, “culture” can point to a group of people, often the inhabitants of a national state, which is supposed to be comparable not only with respect to some patterns of behavior, but also to a putative common ethnicity and biology (i.e., genetic expression and brain function) as well as social heritage (i.e., practices and beliefs) (Han and Northoff, 2008; Chiao, 2010; Han et al., 2012). This rather monolithic concept of culture has been criticized in anthropology for over 30 years.

The aim of the current *Hypothesis and Theory* is to examine the concept of “race” with respect to its alleged scientific usefulness for the classification of humans. We will then discuss and reference alternative concepts and their respective relevance to biological and cultural studies, especially for the assessment of intercultural diversity and the transcultural application of psychiatric research concepts (Heinz and Kluge, 2012).

## The origin of the “race concept” and controversies about its biological usefulness

The concept of “race” has considerably changed through time (Banton, 1987; Wolf, 1994). Related concepts such as the Latin “gens” originally denoted an individual’s “line of decent”, which can entail larger groups; for example classical Latin and Greek texts (e.g., Plinius’ Natural History) in their cosmological schemata divided inhabitants of the world into civilized, barbarian and monstrous groups (Friedman, 1981). In the 15th century, the term “race” (or “raza”) started to be gradually used in European languages; it first appeared at the end of the 15th century at the time of the Spanish Reconquista and was used to describe persons with “Jewish or Moorish heritage”. From the beginning, the term “race” was associated with a negative connotation of static, unchangeable and undesirable heritable characteristics (Hering Torres, 2006; Brückmann et al., 2009; Blum, 2010; for a more detailed historical overview on development of the “race concept” see Friedman, 1981; Banton, 1987; Wolf, 1994). The scientific and popular usage of the concept of “race” increased in the 18th and 19th century. In the context of 18th century natural classification, classifying human variation started to be of increasing interest, just to mention a few: Francois Bernier, Carl Linneaus; Count de Buffon, and J.F. Blumenbach, a German anatomist and anthropologist (Banton, 1987).

“Modern race concepts” and the classification of different “races” can be traced back to Blumenbach. In the 18th century, he suggested that there are no major qualitative differences between the human “races”, which nevertheless differ in their “degree of beauty” (Blumenbach, 1795). At this time, it was widely assumed that God had created mankind in a perfect condition and that the variety in human physiognomy reflected varying degrees of “degeneration”. “Caucasians”, the term that Blumenbach coined for what he considered to be the “European race”, were considered to be the least degenerated and hence, the “most beautiful race of men” (Blumenbach, 1795). Later theories on human diversity had to cope with the concept of evolution, which apparently contradicted the idea of “degeneration of a perfectly created mankind”. Rather, it suggested that the human species developed from its phylogenetically older and more primitive ancestry towards its current state. In the 19th century, several theories suggested that evolution had occurred in different places in the world independently from each other, hence creating the major human “races” (polygeny) (for a critical reflection see Gould, 1981). “Races” would thus resemble different species rather than having to be understood as a stereotypic perception of human differences within one species in different parts of the world.

**Figure 1:**
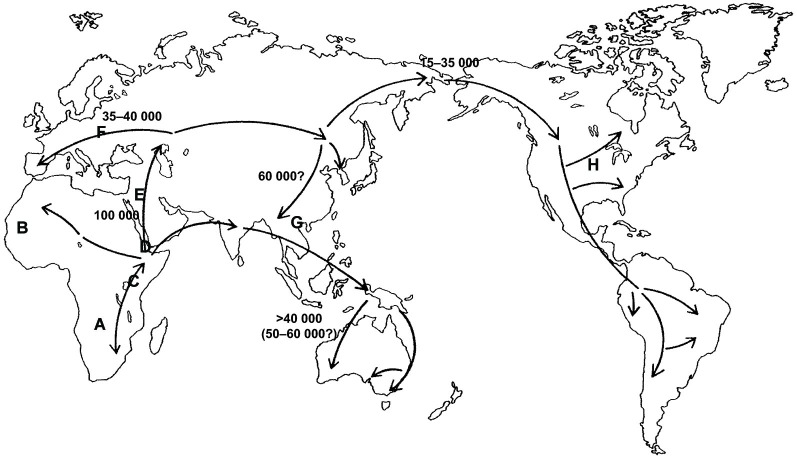
**Hypothetical route of the modern human (*Homo sapiens sapiens*) on his dissemination coming from Africa towards the other continents**. The figures give the proxi dates of the arrival at the different continents (modified based on Cavalli-Sforza and Cavalli-Sforza, 1994: p. 200). The letters A to H represent distributions on regional populations named according to the respective geographical areas. Modified from Heinz et al. (2011a,b: p. 77).

**Figure 2:**
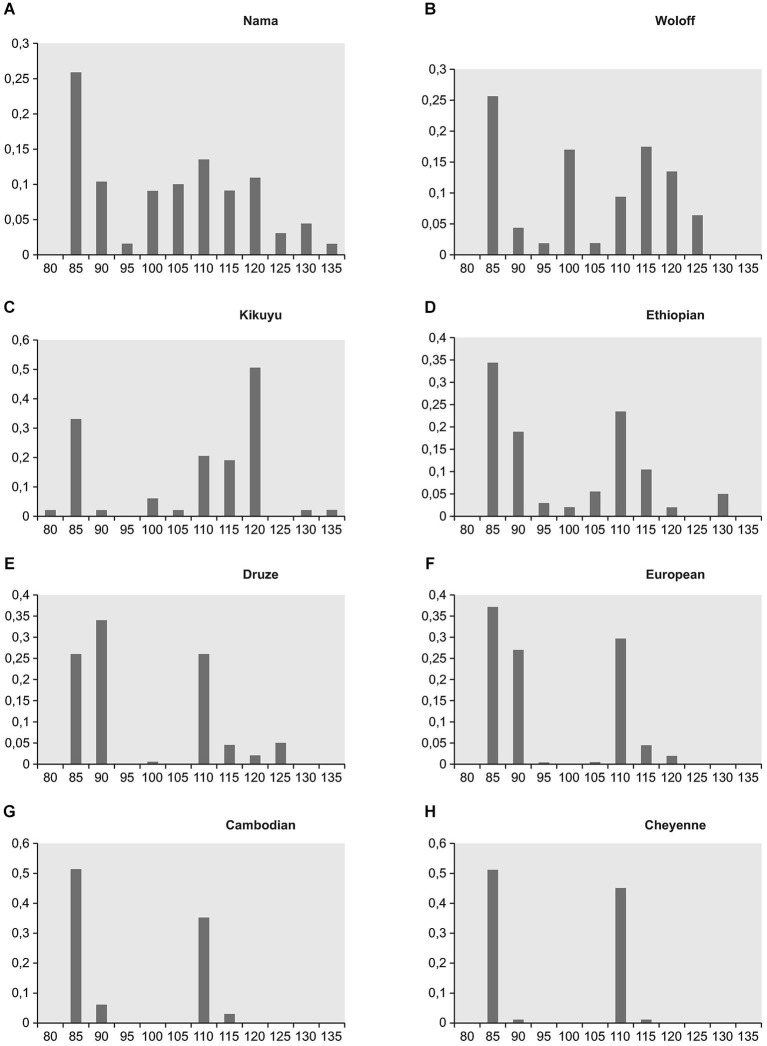
**Frequencies of the different CD–4 haplotypes in four different African populations/regions (A–D) and in four different non-African populations/regions (E–H) (Tishkoff et al., 1996)**. Modified from Heinz et al. (2011a,b: p. 78).

However, current scientific theories discard polygeny, and the “race concept” has been questioned by modern biological research. For example, Livingston argued some 50 years ago that the “race concept” is not useful to classify human diversity, because it postulates categorical gaps between so called “races”, while genetic studies instead suggest a gradual in*cline* or de*cline* of allele frequencies as an indicator of human genetic diversity (Livingston, 1962). This concept is illustrated by a study published in Science in 1996, which showed that the world-wide distribution of single nucleotide polymorphisms and other forms of genetic variations, that constitute different alleles of a gene (and which can be grouped in so-called haplotypes, i.e., specific patterns of allelic variants), support the hypothesis that “modern humans originated in Africa” (Tishkoff et al., 1996). Here, most genetic variants and hence a wide variety of haplotypes of the gene examined in this study were found in Africa. Following the hypothetical route (see Figure 1) of migration towards the Arab Peninsula and then Europe, Asia and ultimately America, haplotype variability is gradually reduced, suggesting that rather small groups of individuals, representing only a limited amount of genetic variants with respect to the original population, migrated to these regions of the world (Tishkoff et al., 1996). The illustration (Figure 2) showing haplotype variants in different world populations (named according to their country of origin) aptly illustrates the concept of de*clining* variability and hence supports Livingston’s claims that modern biologists should speak of *“clines”* instead of “races” and thus address the gradual rather than categorical differences between human populations (Livingston, 1962). Likewise, in their revision of the 1964 UNESCO statement on “race”, the AAPA clearly rejected the “race concept” as inadequate (Association of American Physical Anthropologists, 1996). Humans come in endless diversity, not in limited groups.

## Cultural impact on race classifications

The topicality and problematic nature of the “race concept” is further illustrated by the fact that in everyday life, classifications of groups or populations are based on a mixture of traditional stereotypes about physiognomy and prevalent language patterns as well as current adaptations of originally colonial perspectives (Choudhury and Kirmayer, 2009; Heinz et al., 2011a,b). For example, the so-called “races” used to classify participants at the SfN included the option “Hispanic” or “African American” or “Caucasian”. The label “Hispanic” originates from the previous influence of the Spanish Empire on the official language of their colonies, while the label “African American” was coined to reflect the ancestral origin of migrants, most of whom were not entering the Americas upon their own will, and finally the option “Caucasian” reflects the badly chosen term created by Blumenbach (1795) to denote the supposedly “most handsome and becoming ‘race’ of the world”. The contingency of such classifications can be illustrated by the allocation of Jamaicans. Since Jamaica was conquered by the British in the 18th century, Jamaicans are currently classified as “African Americans”, at least if they fit the stereotypic physiognomic expectations; had Jamaica not been conquered by Britain in the 18th century and instead remained a Spanish colony (e.g., such as Cuba), Jamaicans would today be classified as “Hispanics”.

Worse, one of the consequences of slavery was to create absurdly pseudo-exact classifications (e.g., Röhrbein and Schulz, 1978) denoting the relative percentage of supposedly “inferior” African ancestry, thus promoting a practice that phenotypically categorizes a person as “black” or “African American” as long as he or she matched prejudices about alledgedly typical African phenotypes. This phenotypic assignment persists into today’s social practices and can promote prejudice and discrimination. Moreover, it forces individuals with e.g., a rather dark-skinned mother and a light-skinned father to “take sides”—instead of accepting diversity, human beings are thus forced into the Procrustean bed of racial classifications. Creating “racial” classifications therefore can reify putative differences (e.g., associated with social exclusion) as “biologically based distinctions”—which can then be used in a vicious cycle of racist argumentation to justify the social disadvantage of so-classified people by pointing not to social exclusion but to their alleged biological differences (Lane, 1994; Rothenberg and Heinz, 1998).

The negative social consequences of “racial” classification have been described by Brodkin (2010), who showed that it was not before the end of World War II that Jewish Americans were classified as “white”, which facilitated integration in American society, while previous classifications as distinct from the supposedly homogeneous “white race” presented a massive obstacle to social participation.

Having said this, we would like to emphasize that there is indeed a considerable degree of human genetic variability—however, it does not fall into distinct categories such as “races”. There are no “racial genes” (Fuentes, 2012), there are no “genes” present in one population that are absent in another. Indeed, humans share the same genes but differ from each other in various degrees with respect to the presence of specific allelic variations, with gradual differences in the percentage of certain allelic variations within but no sharp borders between local human populations (Heinz et al., 2011a,b; see Figure 2).

## Racial classifications, colonial hierarchies and the construction of the psychotic patient as primitive man

In the early 20th century, the imagined “racial” hierarchies, with Europeans at the “top” and Africans at the “bottom”, were integrated into an evolutionary construction of mental disorders (for a critical reflection see Heinz, 1998). It was assumed that degenerative or so-called “dissolutive” processes reverse the evolutionary development. Moreover, it was suggested that in any healthy person, their individual development (onthogenesis) is a recapitulation of the development of the species (phylogenesis). While this concept is currently discussed within biology only with respect to intra-uterine development, at the turn of the 19th to the 20th century it was applied also to post-natal development, and it was assumed that infants resemble “savages” or so-called primitive people. Hence, the gradual mental development of children was supposed to recapitulate the phylogenetic development of mankind, and diseases were understood as a loss of the previously acquired and evolutionarily coded level of functioning and a return to a more primitive stage of development (Heinz, 1998). Independent of whether psychiatric theories were mainly developed in a biological or a psychoanalytical context, there was considerable overlap in the construction of psychotic experience—in both instances, it was assumed to reflect a loss of higher cognitive functioning and/or the structural integrity of higher brain centers (which should then clinically manifest as negative symptoms) and a return to a more primitive level of functioning (manifesting as positive symptoms) (Freud, 1912/1913; Jackson et al., 1927).

Since the phylogenetic ancestors of “modern humankind” were unavailable to current observation, psychiatric theories instead examined what they felt to be the best proxy of these supposedly primitive beings—colonialized people. Indeed, there is an inherently racist strain in these theories, which independently of whether the emphasis is laid on psychodynamic or neurobiological development, on “degeneration” or “regression”, assumed that the subjects of colonial rule were incapable of logical thinking and a realistic approach to the environment, and hence a useful example of a “primitive”, irrational level of functioning which was supposedly also observed in psychosis (for an in-depth analysis of this issue see Gould, 1981; Heinz, 1998). This is why Bleuler, in his seminal monograph on schizophrenia (Bleuler, 1988[1911]), claimed that psychotic patients resemble “the Negro” (sic!), who (as Bleuler claimed) is supposed to be as unable to abstain from wishful thinking as any “autistic” schizophrenic patient. In fact, the term “autism” was created by Bleuler as a neologism derived from Freud’s concept of “aut*oerot*ism”. Freud had assumed that in psychosis, there is a regression to a level of early postnatal functioning, in which the main interest is directed towards gaining joy from sucking at one’s own body parts (hence the term of “autoerotism”). Bleuler, somewhat uneasy with the erotic implications of Freud’s theories, simply cut the term “eros” out of “autoerotism” and created the new term “autism”, which as he felt was a good concept to describe both the supposedly unrealistic attitudes of colonialized people in Africa as well as the psychotic state of individuals suffering from schizophrenia (Bleuler, 1988[1911]).

However, when schizophrenia patients and colonialized people were placed on the same imaginary (low) level of human development in the imperial fantasies of European researchers, their positions were equally endangered. Hence, it comes as no surprise that Eugen Bleuler was a fierce proponent of compulsive sterilization in Switzerland and that during Nazi rule in Germany, both schizophrenia patients and the children of French African soldiers occupying the Rhineland after World War I were sterilized against their will (Heinz, 1998). While crude “racial” hierarchies and inadequate identifications of mental disorders with supposedly “lower” levels of phylogenetic functioning are no longer scientifically accepted in present day scientific discourses, the underlying pattern of hierarchical brain functions and their respective loss in psychosis to date confound appropriate conceptualizations of the nature and development of psychotic disorders (Heinz and Schlagenhauf, 2010).

## The social impact of “race” classifications

Despite these concerns, “racial” classifications are still used to justify social differences by pointing, for example, towards supposedly heritable and ethnically determined differences in intellectual capacity as measured in IQ-tests (Herrnstein and Murray, 1996). These tests were carried out despite evidence from adoption studies, which showed that the gap in test performance between so-classified “white” vs. “black” Americans was more than compensated by the adoption into the more socially advantaged group (Weinberg et al., 1992). Furthermore, the dramatic increase in IQ-test performance between 1945 and the mid-1990s (by more than one standard deviation, i.e., about 15 points) suggests that there is a profound environmental effect on average test performance, which is larger than current IQ-test performance differences between various minority and majority groups within American or European populations (Flynn, 1987).

Also, discussions about a genetic causation of allegedly heritable differences between IQ-test performances of different “ethnically” or “racially” classified groups often fail to take into account that any heritability *within* one group does not necessarily explain differences *between* two groups exposed to systematic alterations in their environment. The aforementioned rise in IQ scores during the period between World War II and the 1990s (where IQ test results increased by more than one standard deviation) is much too short to be driven by genetic variations—this massive increase in the ability to solve riddles per minute obviously reflects environmental effects. It also demonstrates that raising IQ test scores does not improve anything beyond IQ test scores; social problems attributed to low IQ test scores by some authors (Herrnstein and Murray, 1996) certainly did not disappear or even decline during this time.

While “races” do not exist, racist discrimination does and can directly affect IQ test scores, as shown by the fact that even brief periods of social exclusion interfere with IQ test results (Baumeister et al., 2002). Differences in IQ test scores between groups classified with biologically nonsensical, but discriminating “race” labels can thus arise as a result of exactly those (racist) classification processes required to compare supposedly homogeneous groups. In spite of these observations, which caution against the reification of social differences as “racially” transmitted heritable traits, popular books published without peer review but addressing large audiences such as “The Bell Curve” (Herrnstein and Murray, 1996) in the nineties or Thilo Sarrazin’s book (Sarrazin, 2011) on the alleged cognitive disadvantage of Turkish migrants in Germany impact on the same social fabric that they try to describe by questioning the ability of socially excluded and discriminated minorities for participation in society.

## “Culture” as a proxy of “race”

In neuroscience, there is an increasing amount of scholars who try to sidestep the issue on “race” by replacing it with the allegedly neutral term “culture” (e.g., Han and Northoff, 2008; Chiao, 2009; Losin et al., 2010; Han et al., 2012). One prominent example in the field of so-called cultural neuroscience is an article by Han and Northoff (2008), who attempt to account for “cultural” differences in brain sizes during spatial normalization in the preprocessing of fMRI data. Thereby, they referred to the work of J.P. Rushton (Rushton and Ankney, 1996) to support their claims that differences in brain size may be attributed to “culture”, unaware (as indicated in an ensuing personal communication) that in Rushton’s work, “culture” directly translates to “race”. In his work, Rushton claimed that there are three major “races” that systematically differ in brain and penis size, IQ and cultural achievement. This example shows that terms such as “culture” are not neutral when used to assess differences between supposedly homogeneous populations in neuroscience (Martínez Mateo et al., 2012).

Indeed, some studies use broad and ambiguous terms like the concept of “cultural identity” (e.g., “the Chinese self” Zhang et al., 2006) and suggest that “culture” is a homogenous category that refers to a supposedly homogeneous ethnic group, which expresses this uniform “culture”, e.g., due to shared sociobiological traits (Taguieff, 1991; Balibar and Wallerstein, 2011; Martínez Mateo et al., 2013a,b). It has been warned that such use of “culture” can be “neo-racism”. The term “neo-racism” illustrates that approaches to “cultural diversity”, despite having overcome a system of “racial” hierarchies, share the idea of clearly definable “cultural group” demarcations that originate from supposedly biological differences (Brown, 1993). For example, the assertion that “Native Chinese and Chinese Americans may be thought to belong to the same racial group but may have distinct cultural values and beliefs and experiences” (Han et al., 2012) is directly based on a racial categorization of groups. In the same article, the authors refer to “race” as “the way of categorizing human beings on the basis of external attributes, such as skin tone and facial and body shapes that differentiate human populations”, thus making the interchangeability of the terms “race” and “culture” obvious. In this respect, the idea of clearly definable “cultural” demarcations cements “cultural” belonging as essential, similar to the biological concept of “race” (Choudhury and Kirmayer, 2009; Martínez Mateo et al., 2012, 2013a,b).

Similarly, in transcultural psychiatry, the term “culture” is often ambiguously defined and interchangeably used to *either* denote language patterns and cultural practices *or* as an inadequate proxy of putatively biological differences between certain “ethnic groups” or “races”. This is evident when “cultural” classifications are applied at the empirical level. For example, does a study of “Turkish women in Germany” actually study only subjects of Turkish nationality living in Germany or also the children of Turkish migrants with some parents born in Turkey, others in Germany? Furthermore, should such a study include only subjects speaking Turkish or any other language currently present in Turkey or does it simply aim at individuals with a Turkish family background (Terkessidis, 2004; Aichberger et al., 2012; Bromand et al., 2012; Heredia-Montesinos et al., 2012)? While language patterns actually appear to influence idioms of distress to a considerable degree, which needs to be reflected in patient-healer interactions (Kleinman, 1981; Penka et al., 2003; Napo et al., 2012; Vardar et al., 2012), “culture” apparently is often used to denote supposedly more profound and potentially biologically rooted differences between groups. The term “culture”, particularly when used to describe individuals originating from certain national states, thus often tends to ignore diversity and to suggest a homogeneity that consciously or unconsciously appears to extend into the realm of biological similarities and differences (Martínez Mateo et al., 2012).

In contrast, anthropologists such as Clifford Geertz act on the assumption of a “process-oriented, semiotic understanding of culture”, where values, beliefs and practices constitute cultural knowledge attained through experience of the social and physical world (Geertz, 1973). There, “culture” is no longer understood as a defined and clearly demarcated entity, but rather refers to a humankind-generated complex of thoughts, perceptions, and values, which are materialized in symbolic systems (Reckwitz, 2000). Therefore, culture is not confined to particular sub-domains but instead incorporates multiple, complex, dynamic, diffuse and hybrid networks (Reckwitz, 2000; Schlehe, 2006): “we are all members of multiple, indeed myriad, groups—crosscutting, overlapping, and ever-evolving” (Wallerstein, 1994).

The term culture has also been discussed controversially in anthropology. For example, Kuper questions the analytic utility of the term and states that the current politicized discourse on “culture” provokes uneasy reflections on the implications of anthropological theory (Kuper, 1999). He concludes that common sense in anthropology tells us to reject the assumptions that those differences are natural, and that cultural identity must be grounded in a primordial, biological identity; instead, he places great emphasis on differences and identity. Michaels (1995) and Kuper (1999) thus question the use of modern cultural concepts, which can be a form of racism, because “cultural identity” is often alternatively used to denote “racial identity”. Indeed, it has long been suggested that biological phenotypes and the respective norms used to classify “races” vary not between racial categories but rather according to local environmental factors and are highly dynamic. Some decades ago, Canguilhem (1974) emphasized this dynamic nature of human physical performance, noting that physiological norms cannot be defined universally by a single standard. For example, across centuries, ever-increasing physical achievements in sports are evidence for the immense range of human physical capability. Other such examples include the “Flynn effect” coined to denote gradually increasing IQ test performance (Flynn, 1987) or variations in stature, which systematically vary across centuries and appear to depend on a variety of factors, including nutrition quality. The anthropological “biocultural approach” emphasizes such effects of the environment on individuals and their biological constitution, stressing the malleability and diversity of human physiological performance (Blakey, 2001).

These concepts have recently been of renewed interest to a contemporary approach in anthropology, which attempts to link anthropology and biomedicine. This approach suggest to reconsider the traditional view on physiological differences and introduces the term “local biologies”, which focuses on the interaction between the environment and human physiology (Lock, 2001). Indeed, human biology is subject to evolutionary changes, which are driven by direct biological as well as more indirect environmental and social factors. Thus, the aim is to overcome the gap between cultural and biological approaches of categorizing individual differences (Lock and Nguyen, 2010). Furthermore, recent advances in epigenetics provide a glimpse of the molecular mechanisms involved in mediating the interaction between environmental factors and the human genome, and help to explain individual variations in genotype effects (Meaney, 2010).

## Implications of cultural and genetic diversity in psychiatry

One may raise the question as to why it would seem important to consider “races” (or “ethnicities” or “cultures”) in the context of psychiatry (note that in this context, “culture” usually refers to anything which is likely not primarily influenced by genetics, but where a given population is sharing to some degree important customs, habits, attitudes, religious views etc.). A straight-forward answer to this question points to the fact that the sociocultural background of a group of individuals can have implications on the prevalence rates and type of psychiatric syndromes, which individuals experience when exposed to specific stressors (e.g., traumatic experiences) in interaction with their variable biological profiles. For example, the rates of alcohol dependence are determined by drinking customs (and tend to decrease in Southern Europe while tending to be higher in Central-Eastern Europe; Rehm et al., 2012) as well as by genetic profiles (e.g., lower rates of alcoholism in carriers of particular ALDH2 gene variants; Müller et al., 2010). In addition, any treatment outcome is also typically affected by cultural customs (e.g., smoking or grapefruit consumption affecting drug metabolism) and genetics (e.g., higher prevalence of HLA-B*15:02 carriers in certain populations in Asia with a risk for serious cutaneous side effects when exposed to carbamazepine; Leckband et al., 2013).

Thus, while the concept of “races” is biologically meaningless and potentially offensive, the knowledge of cultural practices as well as of geographic variations in allele frequencies can deliver helpful and important information in clinical practice (Lee et al., 2001). However, considering the “cultural background” of a person can be complicated and may be highly subjective (e.g., in multinational, migrant families); likewise, studies on current geographic variations in allele frequencies at best identify statistical differences in risk profiles, which are constantly changing due to human migration in a globalized world. Therefore, only individual genetic profiles deliver “stable” and highly reliable information at steadily decreasing costs.

When the Food and Drug Administration (FDA) approved the first drug (i.e., isosorbide dinitrate/hydralazine) only for a group hypothesized to be somewhat “homogenous”, the so-called African-Americans, genetic analyses were not considered to determine which genetic profile may exist that should have resulted in significant success rates *exclusively* in this particular demarcated group, which were not seen in other groups (i.e., the drug is supposed to be ineffective in other putatively “racially” defined groups due to unknown factors). However, today there are several examples where individual genetic profiling can help to predict drug plasma levels (Müller et al., 2013a), response to treatment (Sturgess et al., 2011) or occurrence of side effects (Müller et al., 2013b). It is important to keep in mind that for some populations, the frequencies of such gene variants can vary substantially. For example, for the CYP2D6 gene, which is involved in the metabolism of numerous drugs used in psychiatry, only less than 5% of all subjects currently living in Europe are ultra-rapid metabolizers (UM), while in contrast approximately 40% of today’s inhabitants of North-East Africa are UM (Sistonen et al., 2007). The first individual pharmacogenetic tests incorporating information from multiple gene variants have become available with first data showing promising clinical validity and utility (e.g., Winner et al., 2013). With continuously decreasing costs to obtain genomic information and advances in bioinformatics, medicine has started to enter a new era. Genetics will provide caregivers with precise individual information in order to achieve so-called personalized medicine (a better expression may be individualized medicine, since the approach focuses on individual differences, not personal aspects of a human being). In any case, precise individual information will be substantially more valuable than considering clinical information based on controversial “racial” classifications.

In the interest of each individual patient, clinicians should consider cultural practices (such as grapefruit consumption frequencies) as well as current geographical variations in genetic risk profiles for drug side effects, and they should screen for unusual outcomes to drug treatment in order to perform well-informed diagnostic and therapeutic decisions. However, the use of genomics will likely become an increasingly important complimentary source of information, which as we expect will replace the current categorization of “ethnicity” when used for treatment decision.

## Summary and outlook

In summary, while the controversial term “race” has been deemed as inappropriate by most researchers in the recent past, the term has currently seen a revival in the context of neurobiological and psychiatric studies on “culture”. We caution that the terms *race* and *culture* are used interchangeably, and that both are based on an inappropriate categorization of groups that ignores diversity and reinforces stereotypical thinking and discrimination (Hacking, 1995). Instead, we suggest that “culture” refers to a socially generated and dynamic complex of thoughts, perceptions, values and meanings, which incorporate multiple, diffuse and changing networks. We are well aware of genetic differences between human beings and emphasize that human diversity is inappropriately dissected by categorical classifications into broad, biologically meaningless and historically compromised concepts such as “race”. Instead, we suggest to focus on individual differences and vulnerabilities that interact with social processes such as isolation, exclusion and discrimination in the development of mental disorders (Heinz and Schlagenhauf, 2010; Heinz et al., 2011a,b). We are concerned that a search for “culture-specific” functional or structural neuroscientific patterning can reveal an understanding of “culture” that equals old-fashioned concepts of “races” and still appeals to biology, “blood” and ancestry, indicating a transition from classical to cultural racism (Martínez Mateo et al., 2012).

What returns today in scientific discourse, culturalized concepts of race and racialized concepts of culture, is deeply imbued by its past. There is no neutral and harmless use of the “race” concept. When we speak of the “uncanny” return of the race concept, we refer to the dangerous tradition of social exclusion and discrimination justified by supposedly biological “race” differences. Moreover we want to emphasize that the process of “othering”, which is required to neglect human diversity and to place humans in the Procrustean bed of race categories, has its own spooky consequences. The more we deny or conceal our human emotions, passions and desires when trying to conform to society’s rules, the more we can be tempted to project exactly these unwanted desires on the prototypical “Other” (Freud, 1919), be it the “other race”, “ethnicity” or “culture” as constructed by biologically useless by highly suggestive dichotomies between “us” and “them”. However, “the foreign is the own, familiar, cryptic and secret in the Other as the Other...the uncanny” (Plessner, 2003). What haunts current scientific discourse when using concepts of “race” and “culture” is a tradition of social exclusion and a practice of “othering” that as we caution may alienate us scientists from ourselves as well as from our fellow human beings.

## Conflict of interest statement

The authors declare that the research was conducted in the absence of any commercial or financial relationships that could be construed as a potential conflict of interest.
